# Integrating scRNA and bulk-RNA sequencing develops a cell senescence signature for analyzing tumor heterogeneity in clear cell renal cell carcinoma

**DOI:** 10.3389/fimmu.2023.1199002

**Published:** 2023-07-12

**Authors:** Qiming Gong, Yan Jiang, Junfeng Xiong, Fahui Liu, Jikui Guan

**Affiliations:** ^1^ Department of Pediatric Oncology Surgery, Zhengzhou Key Laboratory of Precise Diagnosis and Treatment of Children’s Malignant Tumors, Children’s Hospital Affiliated to Zhengzhou University, Zhengzhou, China; ^2^ Department of Nephrology, Affiliated Hospital of Youjiang Medical University for Nationalities, Baise, Guangxi, China; ^3^ School of Basic Medical Sciences, Youjiang Medical University for Nationalities, Baise, Guangxi, China

**Keywords:** ccRCC, senescence, single-cell, DUSP1, tumor heterogeneity

## Abstract

**Introduction:**

Cellular senescence (CS) plays a critical role in cancer development, including clear cell renal cell carcinoma (ccRCC). Traditional RNA sequencing cannot detect precise molecular composition changes within tumors. This study aimed to analyze cellular senescence’s biochemical characteristics in ccRCC using single RNA sequencing (ScRNA-seq) and traditional RNA sequencing (Bulk RNA-seq).

**Methods:**

Researchers analyzed the biochemical characteristics of cellular senescence in ccRCC using ScRNA-seq and Bulk RNA-seq. They combined these approaches to identify differences between malignant and non-malignant phenotypes in ccRCC across three senescence-related pathways. Genes from these pathways were used to identify molecular subtypes associated with senescence, and a new risk model was constructed. The function of the gene DUSP1 in ccRCC was validated through biological experiments.

**Results:**

The combined analysis of ScRNA-seq and Bulk RNA-seq revealed significant differences between malignant and non-malignant phenotypes in ccRCC across three senescence-related pathways. Researchers identified genes from these pathways to identify molecular subtypes associated with senescence, constructing a new risk model. Different subgroups showed significant differences in prognosis level, clinical stage and grade, immune infiltration, immunotherapy, and drug sensitivity.

**Discussion:**

Senescence signature markers are practical biomarkers and predictors of molecular typing in ccRCC. Differences in prognosis level, clinical stage and grade, immune infiltration, immunotherapy, and drug sensitivity between different subgroups indicate that this approach could provide valuable insights into senescence-related treatment options and prognostic assessment for patients with ccRCC. The function of the gene DUSP1 in ccRCC was validated through biological experiments, confirming its feasibility as a novel biomarker for ccRCC. These findings suggest that targeted therapies based on senescence-related mechanisms could be an effective treatment option for ccRCC.

## Introduction

Renal cell carcinoma (RCC) is the most typical malignant tumor in the kidney from renal epithelial cells. RCC patients’ five-year overall survival (OS) is below 20% (1). Clear cell RCC (ccRCC) is the most common histology subtype and contributes about 70% to all cases of RCC (2). Surgical excisions are the preferred method of ccRCC treatment. However, the outcome is disappointing and is expected to be recurrent (3). The pathogenesis of ccRCC is influenced by the tumor microenvironment (TME), which includes malignant tumor cells, tumor-associated macrophages (TAMs), CD8 T cells, and fibroblasts (4). Even though many biomarkers have been proposed to assess risk models of ccRCC, a significant insufficient value remains with these models (5). Therefore, developing a new predictive model based on the significant genes and pathways of ccRCC is critically essential.

Cell senescence (CS) is a permanent break in the cell cycle and can be caused by various physiological and pathological situations, including tissue injury, aging, and tumorigenesis (6). CS can suppress the unregulated proliferation of cancer cells, thus inhibiting tumor progression (7). Studies also indicated that CS was a robust prognostic biomarker for many cancers in which senescent cells in tumor tissues may facilitate the proliferation and invasions of adjacent pre-neoplastic cells (8). However, the biological mechanisms and prognostic roles of senescence-related genes remain unclear. There is currently little understanding of whether the characteristics of CS within the ccRCC samples can be used for treatment guidance and screening out the prognostic risk subgroups.

Traditional RNA sequencing (bulk RNA-seq) profiling is conducted on a mixed population of cells, which is insufficient for detecting particular cell types and unable to assess the complexity of intra-tumor heterogeneity (9). In contrast, the single-cell RNA-seq (scRNA-seq) technique has flourished recently, allowing researchers to intuitively identify specific gene characteristics at a genome-wide scale and investigate cellular heterogeneity. In this study, we integrated scRNA-seq and bulk RNA-seq data to analyze senescence-related pathways with “ccRCC characteristics” at multiple levels. Based on the genes in these distinct senescence-related pathways, we constructed senescence-related subtypes and risk models and identified significant differences between different subtypes and risk groups in biology and clinical phenotypes. Additionally, we discovered a novel molecular marker, DUSP1, in ccRCC. These findings highlight the important value of cellular heterogeneity in ccRCC and lay the foundation for further development of clinically relevant applications.

## Materials and methods

### Data acquisition

The scRNA-seq files were collected from GSE159115 via the GEO database. The dataset includes 14 samples. Among them, seven lesions were ccRCC tumor samples, one lesion was a Chromophobe RCC tumor sample, and six lesions were cancer-adjacent normal tissues. For ccRCC clinical phenotype data, 526 ccRCC samples and 72 cancer-adjacent normal samples were downloaded from The Cancer Genome Atlas (TCGA) dataset that matched survival information (survival time and survival status). TCGA ccRCC gene-level copy number variation (CNV) data of Masked Copy Number Segment type assessed using the GISTIC2 method was performed. The predictive value for the risk model was validated using RECA-EU data of 91 ccRCC samples downloaded from the ICGC Data Portal. Furthermore, we utilized the GSE167573 dataset downloaded from the GEO database and the E-MTAB-1980 dataset downloaded from the ArrayExpress database (https://www.ebi.ac.uk/arrayexpress/) to further validate the performance of our risk model. Finally, to validate the model’s application in immunotherapy, we downloaded the datasets GSE78220 and GSE135222, along with their respective clinical information, from the GEO database. Additionally, we obtained the expression matrix and clinical data for IMvigor210 using the R package “IMvigor210CoreBiologies”.

### Single-cell data processing

Data analysis was conducted using Seurat R package (version 3.6.3, https://satijalab.org/seurat/). At first, by setting the criteria that each gene expressed in no fewer than 3 cells and no fewer than 250 genes expressed in each cell, single-cell data were filtered, and 32352 cells were obtained. Then, according to the criteria that each cell expressed 100 to 5000 genes, 25% less mitochondrial content, and 100 to 50000 unique molecular identifier (UMI) counts. The proportions of mitochondria and rRNA were calculated using the PercentageFeatureSet function. The data of the 14 samples were individually normalized using log normalization. Hypervariable genes were screened with the FindVariableFeatures function (variant features were identified based on a variance stabilizing transformation [vst]). Subsequently, we used the canonical correlation analysis (CCA) method to identify the sample batch with the FindIntegrationAnchors function and integrated the 14 samples using the IntegrateData method genes were subjected to scaling using the ScaleData function, and anchors were obtained through principal component analysis (PCA) dimensionality reduction. The condition dim = 25 was set for cell clustering using FindNeighbors and FindClusters functions (Resolution = 0.2). We next performed t-distributed Stochastic Neighbor Embedding (tSNE) for dimension reduction on cells using the RunTSNE function and annotated subpopulations using several classical immune cell markers. Marker genes in subpopulations were screened using the FindAllMarkers function with the logarithm of fold change (log FC) = 0.5, minimal percent of the differentially expressed gene (Minpct) = 0.5, and adjusted p< 0.05 as screening thresholds.

### Construction of molecular subtypes and risk model

We conducted consensus clustering on gene expression profiles using the ConsensusClusterPlus package. We simultaneously utilized the PAM algorithm and “Euclidean” metric distance and performed 500 bootstraps, with 80% of patients in the training set for each bootstrap. With the number of clusters set as 2 to 10, the consensus matrix and the consensus cumulative distribution function (CDF) were analyzed to calculate the optimal clusters. LASSO Cox regression was achieved using the R package glmnet. Then, the R package timeROC was utilized to perform the ROC analysis of RiskScore for prognostic classification.

### Acquisition and quantification of gene sets

31 genes related to cell cycle progression (CCP) and 24 genes related to angiogenesis were obtained from previous studies (10). In addition, we downloaded 27 genes related to the G1/S phase from the KEGG website. To analyze differences in cell cycle scoring, tumor metastasis scoring, inflammatory response scoring, and telomerase scoring among different subtypes, several common gene sets, including HALLMARK_G2M_CHECKPOINT, HALLMARK_EPITHELIAL_MESENCHYMAL_TRANSITION, HALLMARK_INFLAMMATORY_RESPONSE, HALLMARK_HYPOXIA and REACTOME_TELOMERE_EXTENSION_BY_TELOMERASE were downloaded from the GSEA website (https://www.gsea-msigdb.org/gsea/msigdb/). Finally, we obtained relevant gene sets for 10 tumor-related pathways previously reported in the study, involving different cancer aspects (11). All gene sets were calculated using ssGSEA analysis to obtain corresponding scores.

### Cell culture and transfection

Human ccRCC cell line 786-O (KCB200815YJ) were obtained from the Chinese Academy of Sciences (Kunming, China). Cells cultured as described previously (12). Small interfered RNA (Si-RNA) targeted DUSP1 (Shanghai Gene Chem Co., Ltd.) using GV493 vectors (hU6-MCS-CBh-gcGFP-IRES-puromycin) to silence the DUSP1 gene expression in 786-O cell.

### Colony formation, transwell migration, invasion, Western blot assay

The Colony Formation, Transwell Migration, and Invasion assay of 786-O cell were performed as described previously (13). A minimum of five random fields of view were immediately captured on the 786-O cell. For western blot assay, 786-O cell were incubated with anti-DUSP1 (Cat# AF5286, Affinity Biosciences), anti-β-tubulin (Cat# T0023, Affinity Biosciences), anti-p21 (Cat# AF6290, Affinity Biosciences) and anti-p53 (Cat# AF0879, Affinity Biosciences), following are the specific methods shown (14).

### SA-β-galactosidase detection assay

The activity of SA-β-gal with 786-O cell was performed in accordance with the manufacturer guidelines (C0602, Beyotime Biotechnology, Shanghai, China). The stained cells were visualized under an inverted microscope.

### Statistical analysis

Apart from the stated bioinformatic methods, R (version 4.1.0, www.r-project.org) and GraphPad Prism 8.0 were used to analyze this research. Spearman’s rank correlation was utilized to evaluate the connection between two continuous variables. The relevance of two group divergences was tested using Student’s t-test. *p*< 0.05 was statistically significant.

## Results

### Single-cell clustering and dimension reduction analysis

After filtering, a total of 27,300 cells were obtained. As shown in **Figure S1A**, the UMI count was significantly related to the number of mRNAs, while the amount of UMI/mRNAs was insignificantly linked to the number of mitochondrial genes. **Figures S1B, C** represents the violin plot before and after quality control. Next, tSNE was performed on 27300 cells for dimension reduction using the RunTSNE function, and 12 subpopulations were identified and annotated using several classical immune cell markers (**Figure S2**). Among these subpopulations, subpopulation 4 was T cell (CD2, CD3D, CD3E, CD3G); subpopulation 10 was B cell (CD79A); subpopulation 11 referred to Mast cell (TPSAB1 and CPA3), subpopulation 1 referred to Macrophage (CD163, CD68, CD14); subpopulations 3 and 9 referred to Fibroblast (ACTA2, PDGFRB, NOTCH3); subpopulations 0, 5, 6, and 8 referred to ccRCC (CA9); subpopulations 2 and 7 referred to Endothelial cell (KDR, PECAM1, PLVAP, PTPRB, VCAM1).

**Figure 1:**
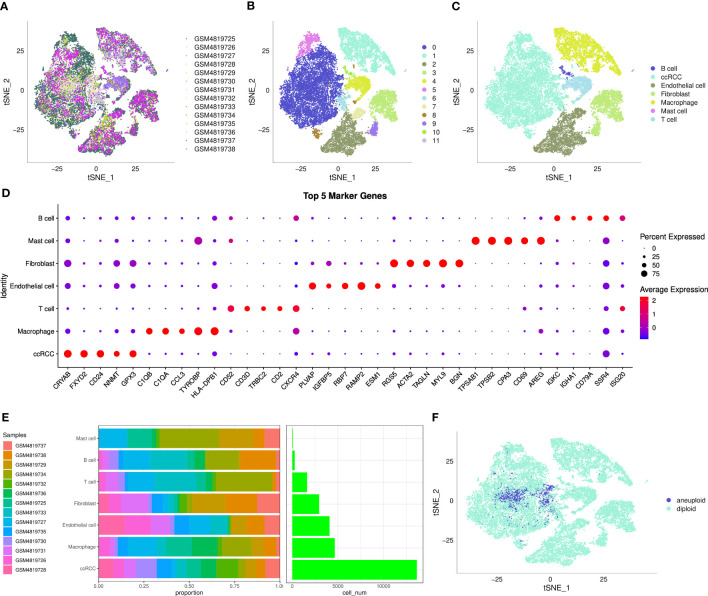
The single-cell landscape of clear cell renal cell carcinoma. **(A)** t-SNE plot showing the distribution of 14 samples; **(B)** t-SNE plot showing the distribution of subclusters after clustering; **(C)** t-SNE plot showing the distribution of cells after annotation; **(D)** dot plot showing the expression of the top 5 marker genes in annotated subclusters; **(E)** proportion and the number of cells in annotated subclusters across samples; **(F)** distribution of malignant and non-malignant cells predicted by copykat.

The distribution of the 14 samples was summarized in a tSNE plot (**Figure 1A**); different subpopulations after clustering were presented in a tSNE plot (**Figure 1B**); the distribution of the annotated cells was visualized in a tSNE plot (**Figure 1C**). The numbers of different sample cells before and after filtering are statistically summarized in **Table 1**. Marker genes in 7 subpopulations were selected using the FindAllMarkers function with logFC = 0.5 and minpct = 0.5. After screening with a corrected p-value< 0.05, we only presented the expression patterns of the top 5 marker genes exhibiting the most significant contribution in the subpopulations (**Figure 1D**). The results of the marker genes are described in Table scRNA_marker_gene.txt. Furthermore, we analyzed the percentages of these 7 subpopulations in each sample (**Figure 1E**). Next, the CopyKat package was employed to predict CNV changes in cells in single-cell data to distinguish tumor cells from normal cells in each sample (although tumor and normal tissues were selected at the time of sampling, it cannot be guaranteed that tumor tissues did not contain normal cells). Among them were 1944 cancer cells and 25356 normal cells (**Figure 1F**).

**Table 1:** Statistics of cell numbers before and after sample filtration.

Sample	raw_count	clean_count	Percentage(%)
GSM4819725	1704	1565	91.84
GSM4819726	1410	328	23.26
GSM4819727	1660	1357	81.75
GSM4819728	923	269	29.14
GSM4819729	3674	3360	91.45
GSM4819730	1097	754	68.73
GSM4819731	578	475	82.18
GSM4819732	2853	2236	78.37
GSM4819733	1644	1366	83.09
GSM4819734	2872	2854	99.37
GSM4819735	1896	1258	66.35
GSM4819736	1953	1787	91.5
GSM4819737	6453	6108	94.65
GSM4819738	3635	3583	98.57

### Cell senescence characteristics in single-cell level

**Figure 2:**
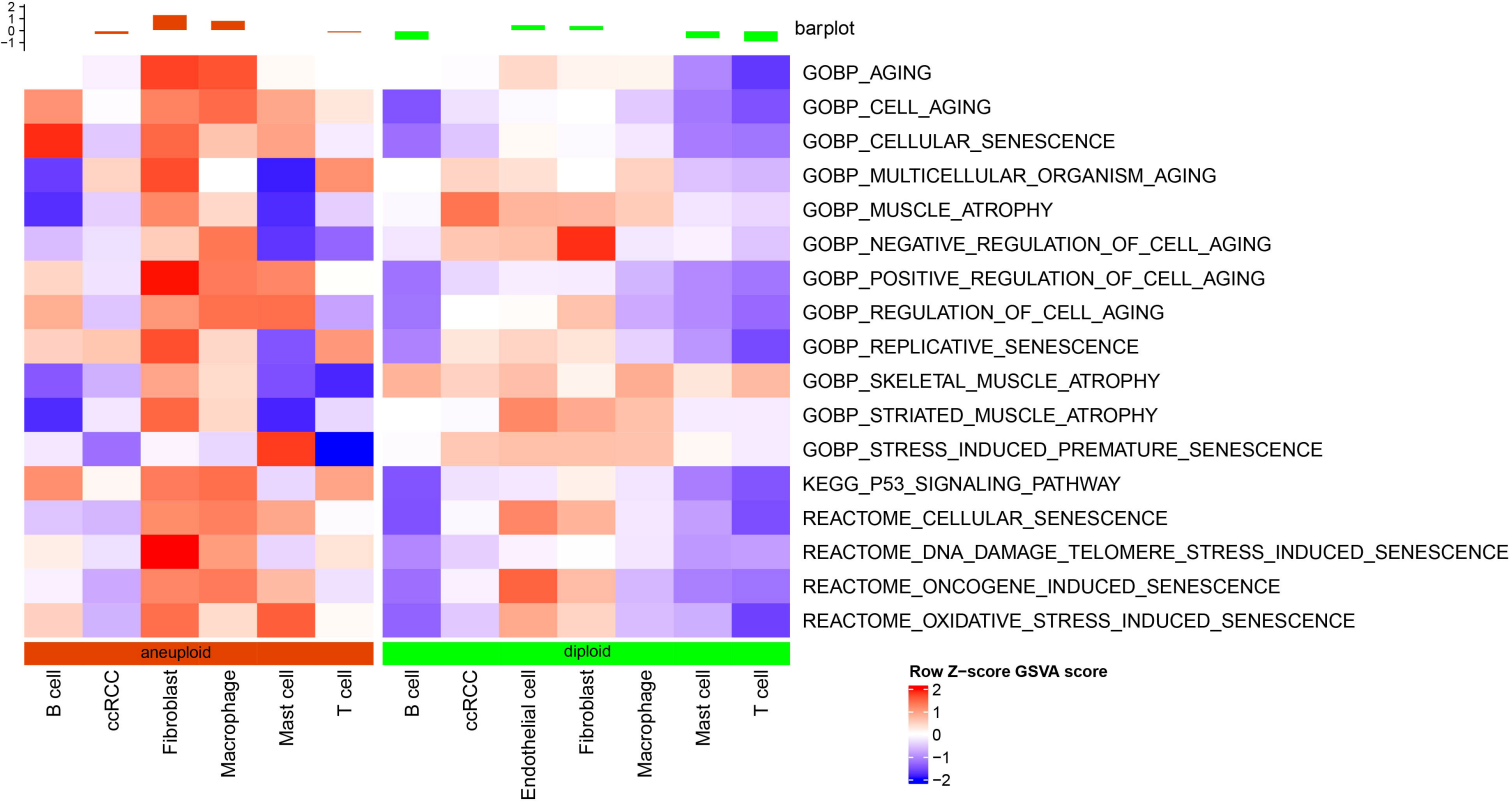
Differential analysis of senescence-related pathways at the single-cell level based on GSVA analysis.

As shown in **Figure 2**, fibroblast senescence-related pathways had higher scores in malignant cells than in non-malignant cells, and endothelial cells were only present in non-malignant cells.

### Validation of cell senescence abnormalities based on bulk RNA-seq data

**Figure 3:**
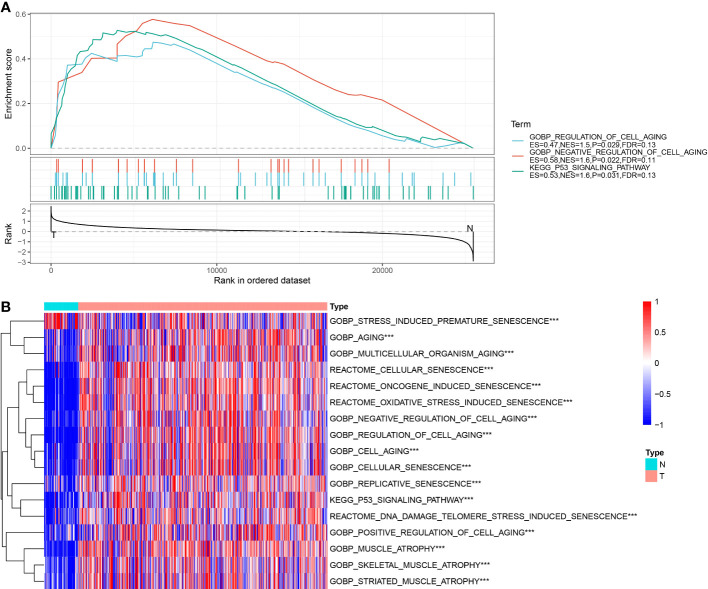
Enrichment results of senescence-related pathways in TCGA dataset. **(A)** Results of GSEA enrichment analysis on TCGA data; **(B)** Heatmap showing expression of senescence-related pathway ssGSEA scores in tumor and adjacent normal tissues of TCGA dataset. ***, p<0.001.

Our results demonstrate that malignant cells exhibit higher expression of cell senescence-associated pathways than non-malignant cells at the single-cell level. We examined expression profiles in tumor and normal tissue samples using bulk RNA-seq data to further analyze these pathways. Through GSEA software analysis, we found that GOBP_REGULATION_OF_CELL_AGING, GOBP_NEGATIVE_REGULATION_OF_CELL_AGING, and KEGG_P53_SIGNALING_PATHWAY were significantly enriched in tumor tissues from the TCGA dataset (**Figure 3A**).

Next, the scores of these senescence-associated pathways in tumor tissues and normal tissues in each sample in the TCGA dataset were subjected to a ssGSEA analysis. The significance of each cell senescence-associated pathway in tumor tissues and adjacent tumor tissues was calculated, and we found the enrichment scores of GOBP_REGULATION_OF_CELL_AGING, GOBP_NEGATIVE_REGULATION_OF_CELL_AGING, KEGG_P53_SIGNALING_PATHWAY was higher in tumor tissues than in tumor-adjacent tissues (**Figure 3B**). The scores of the pathways mentioned above in the TCGA dataset are presented in Table tcga.cellage.score.txt.

### Construction of cell senescence-related subtypes

As described above, our analysis revealed three cell senescence-related pathways, including (GOBP_REGULATION_OF_CELL_AGING, GOBP_NEGATIVE_REGULATION_OF_CELL_AGING, and KEGG_P53_SIGNALING_PATHWAY) were significantly enriched in tumor tissues. Therefore, a univariate Cox analysis was implemented on the genes in these three pathways in the TCGA and ICGC datasets using the survival package and screened prognosis-associated genes with p< 0.05. The results showed 57 prognosis-associated genes in the TCGA dataset and 18 prognosis-associated genes in the ICGC dataset. Furthermore, we found that 7 of these genes were correlated with prognosis in both datasets. The results of univariate Cox analysis on the genes in three senescence-related pathways in TCGA and ICGC datasets are outlined in Table tcga.cellage.cox.txt and icgc.cellage.cox.txt.

**Figure 4:**
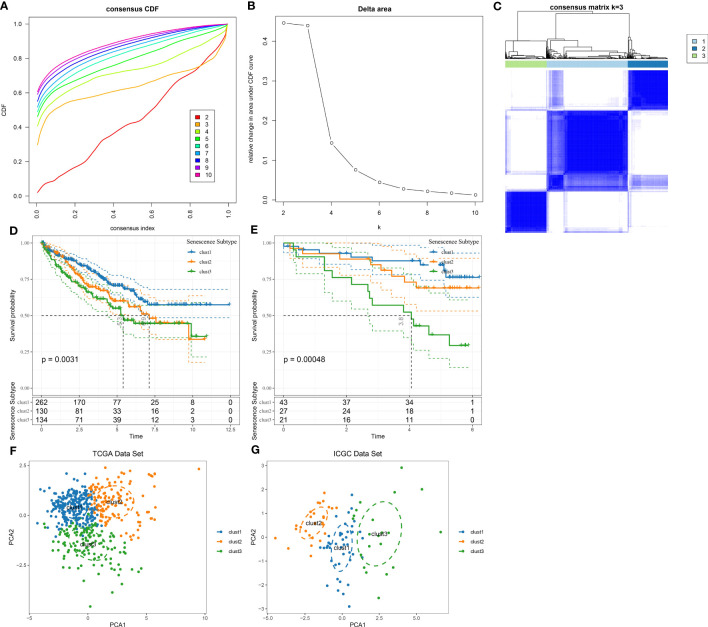
Identification of senescence-related subtypes in ccRCC. **(A)** CDF curve of TCGA cohort samples; **(B)** Delta area curve of consensus clustering for TCGA cohort samples, indicating the relative change in area under the cumulative distribution function (CDF) curve for each category number k compared with k-1. The horizontal axis represents the category number k, and the vertical axis represents the relative change in area under the CDF curve; **(C)** Sample clustering heatmap at consensus k=3; **(D)** KM curves of three subtypes for prognosis in TCGA cohort; **(E)** KM curves of three subtypes for prognosis in ICGC cohort; **(F)** PCA analysis based on senescence-related genes in TCGA dataset; **(G)** PCA analysis based on senescence-related genes in ICGC dataset.

Next, based on seven prognostic genes, we performed clustering using the ConsensusClusterPlus package on 526 ccRCC samples from the TCGA dataset. CDF Delta area curves show that the clustering with Cluster = 3 was more stable (**Figures 4A, B**). Ultimately, we selected k = 3 and obtained three subtypes (cluster) (**Figure 4C**). A subsequent analysis on the prognostic characteristics of these three subtypes was conducted, the results of which revealed that these three subtypes had notable differences in prognoses (**Figure 4D**). Overall, the clust1 subtype exhibited the best prognosis, followed by the clust2 subtype (second) and clust3 subtype (worst).

Additionally, we used the same method to analyze the patients in the ICGC dataset and observed marked differences in prognoses among these three molecular subtypes (**Figure 4E**), which coincided with the results of the TCGA dataset. The results above suggested the transferability of the three molecular subtypes based on feature scores in different study cohorts. The TCGA and ICGC datasets subtypes are presented in Table tcga.subtype.txt and icgc.subtype.txt. Meanwhile, we further conducted PCA on 7 prognosis-related genes in cell senescence-associated pathways. As depicted in **Figures 4F, G**, the PCA results supported that the molecular subtypes constructed based on cell senescence-associated genes were stable and reliable.

### Differential analysis of clinical phenotypes in cell senescence-related subtypes

**Figure 5:**
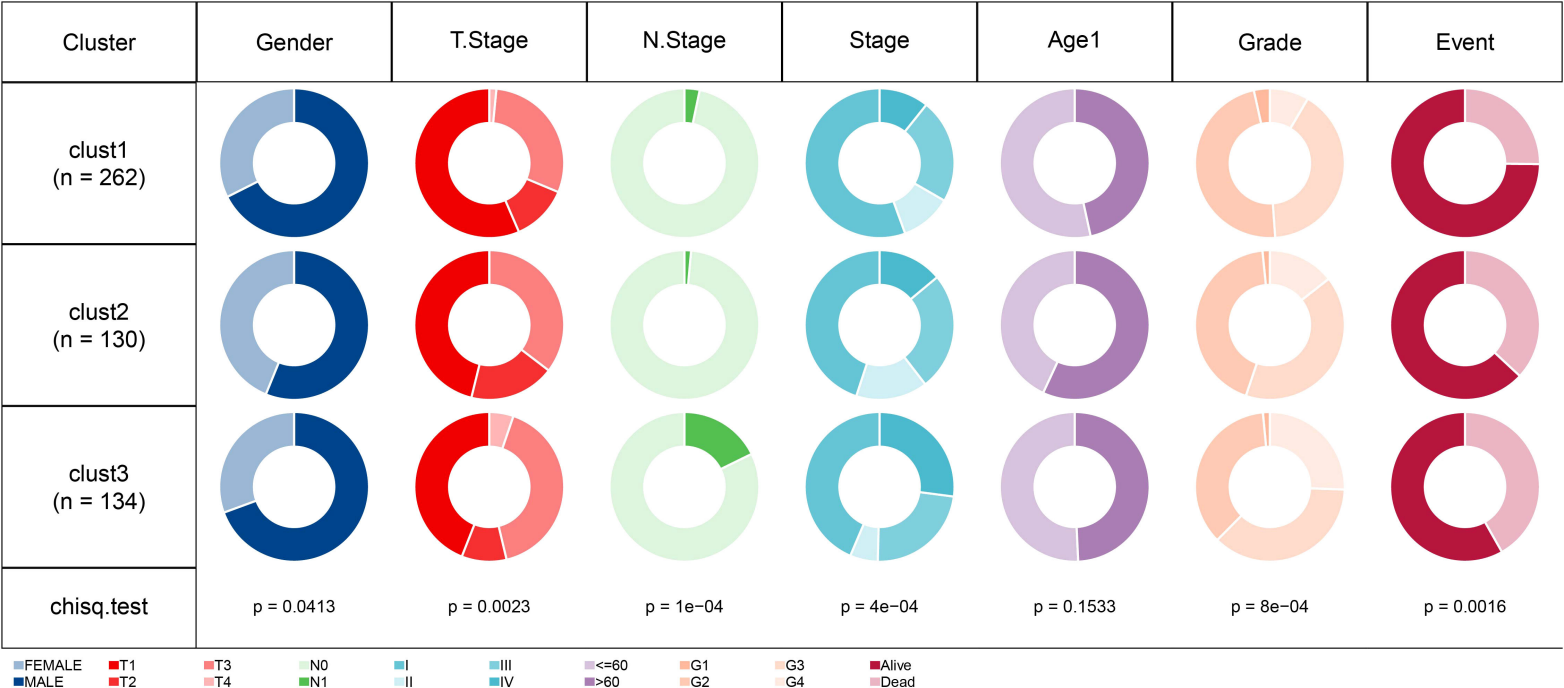
Comparison of different clinical features distribution among three molecular subtypes in TCGA dataset.

For the patients in the TCGA dataset, the distribution of various clinical features in the three molecular subtypes was compared. The corresponding results demonstrated significant differences among the three subtypes concerning gender, T stage, N stage, Stage, Grade, and survival status of patients in the TCGA dataset (**Figure 5**). Additionally, we performed a comparative analysis of various clinical features among the three molecular subtypes in 91 ccRCC patients from the RECA-EU dataset. The results of our analysis demonstrated significant differences in the T stage and nearly significant differences in the M stage among the three subtypes. However, no significant differences were observed in the N stage grouping. (**Supplementary Table 1**).

### Differences in variations of cell senescence-related subtypes

**Figure 6:**
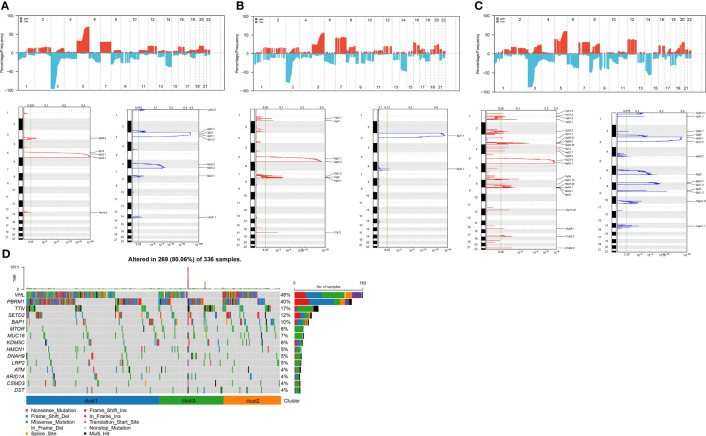
Copy number variation landscape among different subtypes. **(A)** Peak plot of gene copy number amplifications (in red) and deletions (in blue) in clust1 subtype.; **(B)** Peak plot of gene copy number amplifications (in red) and deletions (in blue) in clust2 subtype.; **(C)** Peak plot of gene copy number amplifications (in red) and deletions (in blue) in clust3 subtype.; **(D)** Waterfall plot of the top 15 genes with the most SNV mutations among subtypes.

We next integrated copy-number variants (CNVs) in TCGA-KIRC using the gistic2 software under a confidence level of 0.9, with hg38 as the reference genome. As presented in **Figures 6A–C**, differences were noted in CNVs among the three subtypes. Also, the maftools package was employed to analyze the single nucleotide variant (SNV) data downloaded from TCGA, from which the top 15 genes with the most variations were selected and visualized (**Figure 6D**).

### Biological characteristics of cell senescence-related subtypes

**Figure 7:**
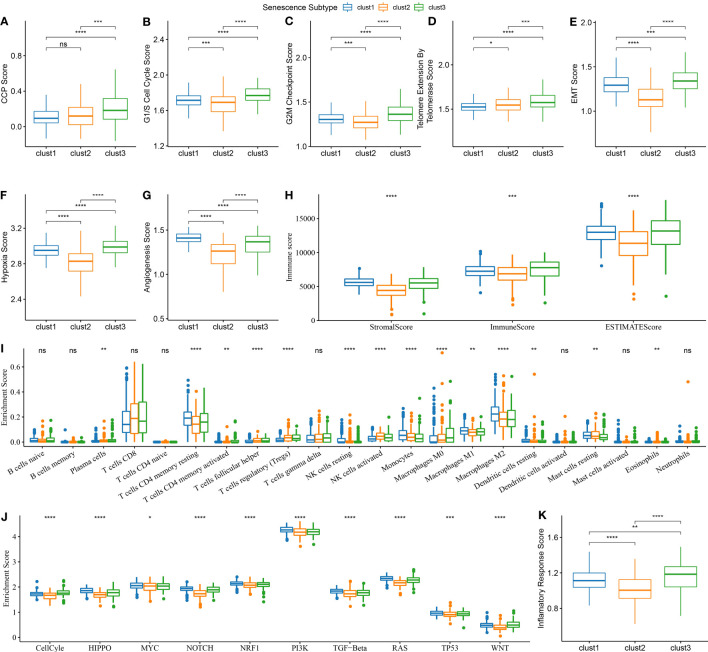
Biological features comparison between different aging subtypes in ccRCC from TCGA dataset. **(A)** Comparison of CCP scores among three subtypes in TCGA dataset; **(B)** Comparison of G1/S phase scores among three subtypes in TCGA dataset; **(C)** Comparison of G2M checkpoint scores among three subtypes in TCGA dataset; **(D)** Comparison of telomerase activity scores among three subtypes in TCGA dataset; **(E)** Comparison of EMT scores among three subtypes in TCGA dataset; **(F)** Comparison of hypoxia scores among three subtypes in TCGA dataset; **(G)** Comparison of angiogenesis scores among three subtypes in TCGA dataset; **(H)** Comparison of immune scores calculated by ESTIMATE among three subtypes in TCGA dataset; **(I)** Comparison of 22 immune cell scores calculated by CIBERSORT among three subtypes in TCGA dataset; **(J)** Comparison of tumor-related pathway scores among three subtypes in TCGA dataset; **(K)** Comparison of inflammation scores among three subtypes in TCGA dataset. ns, p≥0.05; *, p< 0.05; **, p<0.01; ***, p<0.001; ****, p<0.0001.

The CCP score in each sample from the TCGA dataset was calculated using the ssGSEA method. The results indicated that the clust3 subtype, which had the worst prognosis, exhibited a higher CCP score. (**Figure 7A**). Previous research has demonstrated that tumor cells can suppress the induction of cell senescence in the cell cycle, and an essential characteristic of senescent cells is that upregulation of cyclin-dependent kinases such as INK4a and p21 can lead to cell cycle arrest (15).. It is noteworthy that the results also demonstrated an increase in G1/S phase- and G2 checkpoint-related scores in the clust3 subtype (**Figures 7B, C**). These data indirectly illustrated that the cell cycle was not the only influencing factor for cell senescence, and other mechanisms in the body may act together with the cell cycle to regulate cellular senescence.

Likewise, telomerase inhibition induces cellular senescence (15). Tumor cells often activate the telomerase activity to prevent the loss of telomeres in the body. Telomere Extension by Telomerase was the primary function of this pathway. The results of this analysis indicate that the clust3 subtype with a worse prognosis had a higher score of telomere extension by telomerase (**Figure 7D**). Nevertheless, apart from transmitting a “please kill me” message, factors secreted by senescent cells can impact adjacent cells, thus hastening tumor migration and metastasis by inducing epithelial-mesenchymal transition (EMT). Meanwhile, senescent tumor cells can expedite the formation of blood vessels and lymphatic vessels by recruiting specific macrophages and also supply oxygen and nutrients for the growth of other tumor cells, thereby facilitating tumor growth and metastasis. The results showed a higher EMT score of cluster 3 (**Figure 7E**). Furthermore, clustering analysis revealed that angiogenesis and hypoxia scores were significantly lower in the second cluster of patients (**Figures 7F, G**). Next, the immune score and stromal score of samples from TCGA were estimated using the ESTIMATE method. According to these two scores, a higher degree of immune infiltration was noted in the clust3 subtype (**Figure 7H**). The CIBERSORT method scored 22 immune cells from the TCGA dataset. It was found that cell senescence-associated subtypes significantly differed among some immune cells. Macrophages, in particular, exhibit a notable variance in their level of infiltration across distinct senescence subgroups (**Figure 7I**). We scored the enrichment of 10 tumor-related pathways in samples from TCGA-KIRC and observed significant differences in all 10 pathways (**Figure 7J**). Notably, the inflammation score of patients in the clust3 subtype was significantly higher than that of patients in the clust1 and clust2 subtypes, as shown in the clustering analysis results (**Figure 7K**). These findings suggest a potential correlation between the molecular subtype of ccRCC and the TME, which may have implications for personalized treatment strategies in the future.

### Construction and validation of cell senescence-related risk model

**Figure 8:**
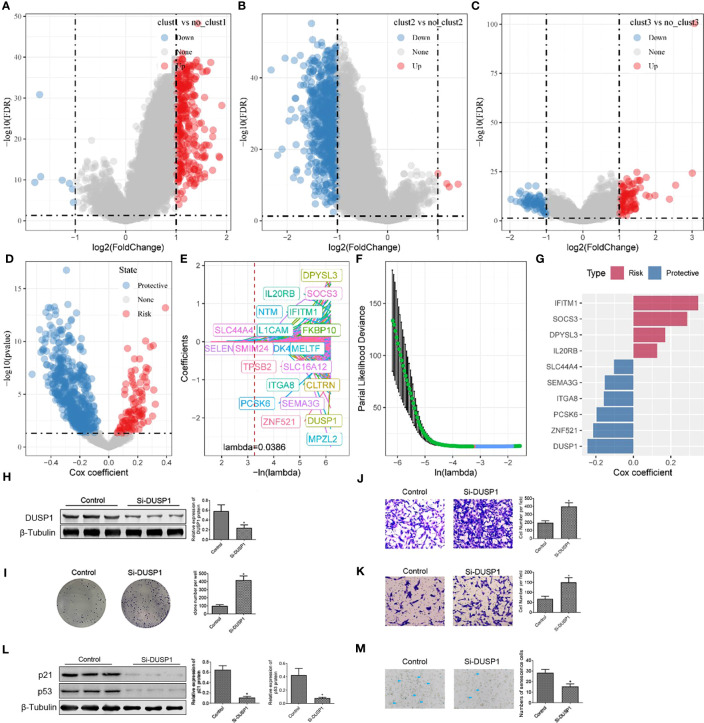
Construction of risk model and functional validation of critical genes. **(A)** Differential analysis between clust1 and no_clust1 in TCGA dataset; **(B)** Differential analysis between clust2 and no_clust2 in TCGA dataset; **(C)** Differential analysis between clust3 and no_clust3 in TCGA dataset; **(D)** A total of 961 promising candidates were identified among the differentially expressed genes; **(E)** Trajectory of each independent variable as lambda changes; **(F)** Confidence interval under lambda; **(G)** Coefficients of prognostic-related genes obtained from multivariate Cox analysis; **(H)** Western blot assay of DUSP1 protein expression in 786-O cell after transfection of Si-DUSP1; **(I)** Colony formation assay was carried out to evaluate the proliferation of 786-O cell; **(J, K)** Transwell assay was used to assess the migration and invasion of 786-O cell. **(L)** Western blot assay of p21 and p53 protein expression in 786-O cell after transfection of Si-DUSP1. *, p< 0.05; **(M)** SA-β-gal staining of 786-O cell. *, p< 0.05.

As described above, we identified three different molecular subtypes through 7 essential genes and found differences in clinical phenotype, mutation, and immune characteristics among subtypes. The clust3 subtype showed the worst prognosis, followed by the clust2 subtype, while the clust1 subtype exhibited the optimal prognosis. Then, we conducted differential analyses of the clust1 vs. no_clust1 subtype, clust2 vs. no_clust2 subtype, and clust3 vs. no_clust3 subtype with the limma package, the results of which are summarized in Table tcga.diff.clust1.txt, tcga.diff.clust2.txt, and tcga.diff.clust3.txt, respectively. Here, we screened differentially expressed genes (DEGs) with p< 0.05 and |log2 (Fold Change)| >1 as thresholds. Ultimately, 314 up-regulated genes and 7 down-regulated genes were found in clust1 vs. no_clust1; 4 up-regulated genes and 754 down-regulated genes were identified in clust2 vs. no_clust2; 95 up-regulated genes and 71 down-regulated genes were obtained in clust3 vs. no_clust3. Lastly, we obtained 964 DEGs for further analysis, as listed in Table all.diff.gene.txt. The results of differential analysis of the clust1 vs. no_clust1 subtype, clust2 vs. no_clust2 subtype, and clust3 vs. no_clust3 subtype are visualized in volcano plots (**Figures 8A-C**).

Next, a univariate Cox analysis of the 964 DEGs was realized using the coxph function of the survival package and identified 613 genes showing significant effects on prognosis (p< 0.05), consisting of 100 “risk” genes and 513 “protective” genes (**Figure 8D**). The corresponding results are summarized in Table tcga.cox.txt. Furthermore, a LASSO regression analysis on the 613 essential genes was implemented to reduce the number of genes for the risk model. The trajectory of each independent variable was analyzed. It was suggested that number of independent variables with a coefficient close to 0 increased gradually as the lambda increased gradually (**Figure 8E**). We employed 10-fold cross validation for model construction and analyzed the confidence interval for each lambda (**Figure 8F**). It should be noted that the optimization model was developed using lambda = 0.0386. Therefore, we selected 21 genes at lambda = 0.0386 as target genes for subsequent analysis. Following stepwise regression, the number of genes was reduced from 21 to 10. Ultimately, a 10-gene signature including ITGA8, SEMA3G, DPYSL3, IFITM1, ZNF521, SOCS3, PCSK6, DUSP1, SLC44A4, and IL20RB was developed, and Senescore was calculated using the formula:


$$\begin{array}{l} \text{Senescore}=-0.158*\text{ITGA}8-0.153*\text{SEMA}3\text{G}+0.17\\ {}*\text{DPYSL}3+0.347*\text{IFITM}1-0.215*\text{ZNF}521+0.288*\text{SOCS}3\\ -0.196*\text{PCSK}6-0.245*\text{DUSP}1-0.103*\text{SLC}44\text{A}4+0.127*\text{IL}20\text{RB}. \end{array}$$

The role of DUSP1 in ccRCC is shown in **Supplementary Figure 1**. To deeper understanding the function of DUSP1 in 786-O cell development, Si-RNA were preformed to silence the expression of DUSP1 in 786-O cell. We found that cells transfected with Si-DUSP1 significantly decreased the expression of DUSP1 protein compared with the control (**Figure 8**). Colony formation assay was used to evaluate cell proliferation. The count of colonies established indicated that the proliferation ability of 786-O cell was activated when transfected with Si-DUSP1, indicating that inhibition of DUSP1 promote 786-O cell proliferation (**Figure 8I**). Transwell assay showed that DUSP1 knockdown increased the number of 786-O compared with the control (**Figures 8J, K**). we identified that knockdown of DUSP1 increased the proliferation, migration, and invasion of 786-O cell. Furthermore, we detected decreased expression of p21 and p53 proteins (canonical protein targets of Cell Senescence), in 786-O cell with suppression of DUSP1 (**Figure 8L**). We also stained for senescence-associated β-galactosidase (SA-β-Gal), a commonly accepted marker for senescent cells. The number of SA-β-Gal 786-O cell was decreased in Si-DUSP1 group compared to control (**Figure 8M**), suggesting that knockdown of DUSP1 suppress cellular senescence of 786-O cell.

### Prognostic analysis and validation of the risk model

**Figure 9:**
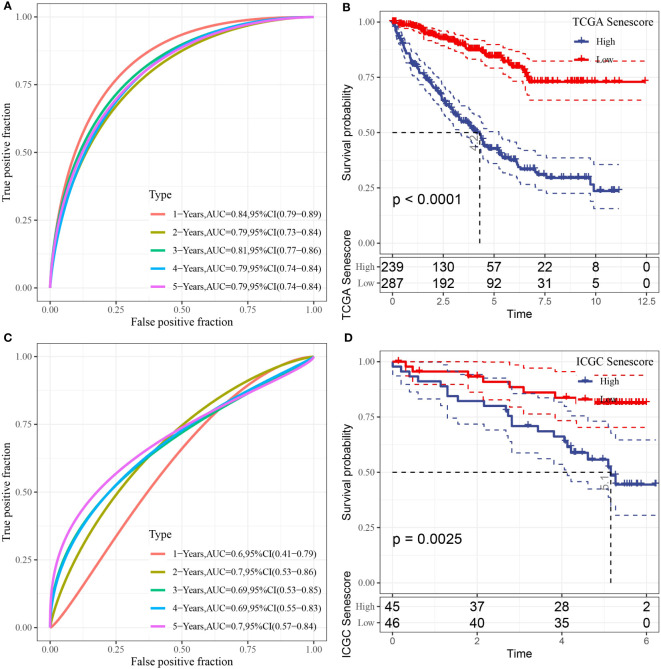
Prognostic analysis and validation of the risk model. **(A)** ROC curve of the risk model constructed by 10 genes in TCGA dataset; **(B)** KM curve of the risk model in TCGA dataset; **(C)** ROC curve of the risk model constructed by 10 genes in ICGC dataset; **(D)** KM curve of the risk model in ICGC dataset.

Next, we calculated the risk score for each sample individually based on the expression profiles of samples in the training dataset from TCGA data. We separately analyzed the classification efficiency of this model to predict 1-year to 5-year prognoses. As depicted in **Figure 9A**, this model had a high area under the curve (AUC) value; At last, we performed Z-score normalization on the Riskscore and assigned the samples to a high-risk subgroup (Riskscore > 0 after Z-score normalization) and low-risk subgroup (Riskscore< 0 after Z-score normalization). Kaplan-Meier (KM) curves were plotted accordingly. The analysis indicated significant differences between the two groups in terms of overall survival, disease-specific survival, and progression-free survival in TCGA-KIRC cohort (**Figure 9B**, **Supplementary Figures 2E–G**). To validate the robustness of the model, the same method was applied for validation using the ICGC dataset. The AUC values of the risk model established with the 10 genes mentioned above are presented in **Figure 9C**. After the Z-score normalization of Riskscore. The samples were assigned to the high-risk subgroup and low-risk subgroup. The KM curves showed significant differences between these two groups in the ICGC dataset (p< 0.05). Furthermore, we validated the robustness of our model in two additional microarray datasets, as shown in **Supplementary Figures 2A-D** by the Kaplan-Meier (KM) curves and AUC values based on the risk model established using the 10 genes. The results indicated that the risk model established using the 10 genes can be effectively applied to microarray data, further confirming the reliability of our research findings.

### Correlations between risk model and clinical characteristics

**Figure 10:**
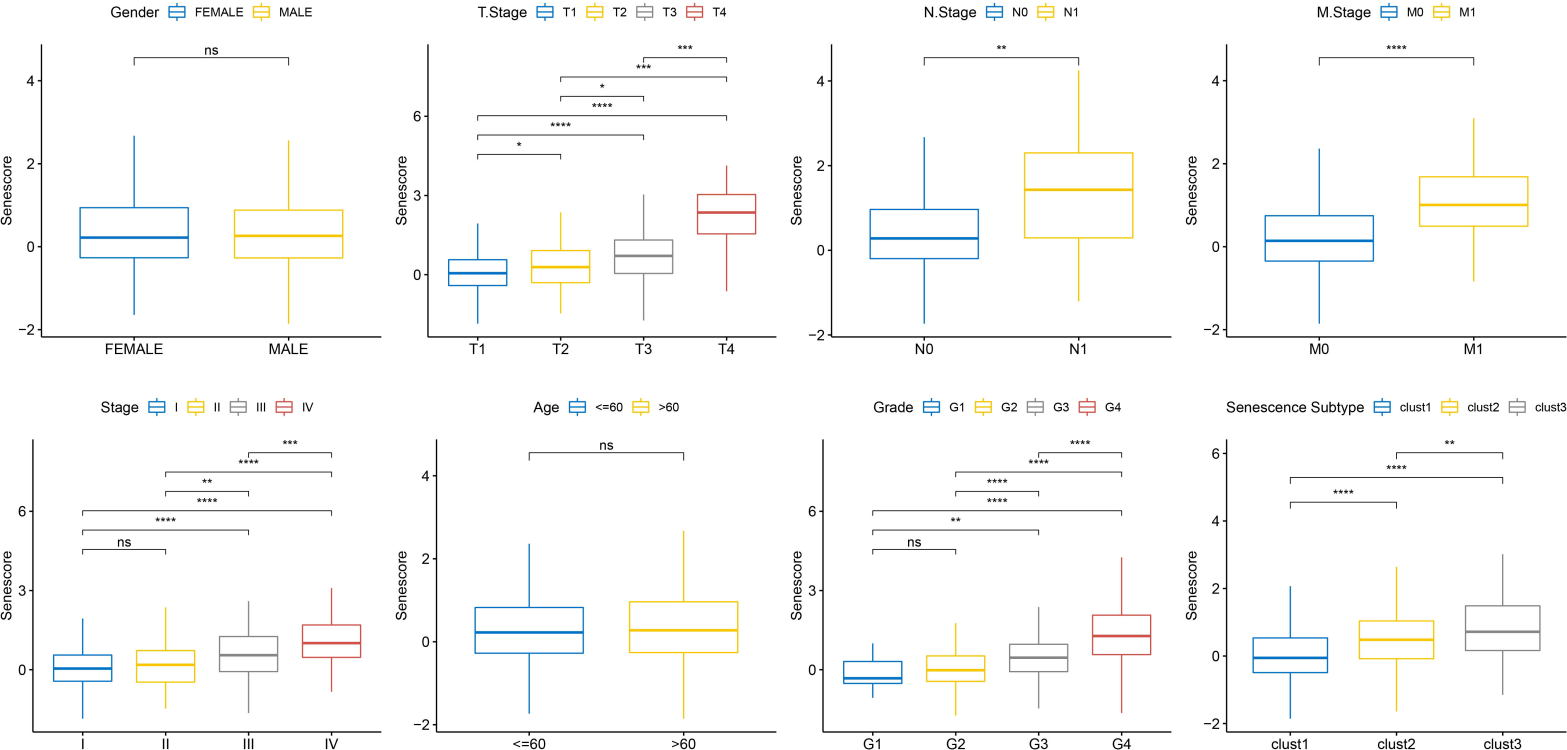
Senescore differences in clinical pathological features in TCGA dataset. ns, p≥0.05; *, p< 0.05; **, p<0.01; ***, p<0.001; ****, p<0.0001.

To ascertain the correlations between the RiskScore and the clinical characteristics of ccRCC, we analyzed the differences in the RiskScore among different TNM grades and Stages in the TCGA-KIRC dataset. The results exhibited that a higher clinical grade was associated with a higher risk score (**Figure 10**).

### Biological characteristics of cell senescence-related risk score

**Figure 11:**
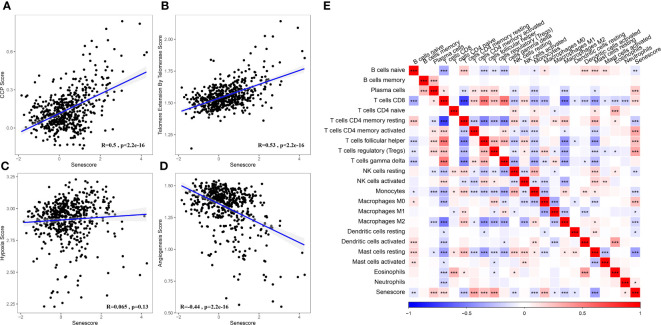
Association between the Senescore and biological feature scores. **(A)** The correlation between CCP score and Senescore. **(B)** The correlation between telomere extension score of telomerase and Senescore. **(C)** The correlation between hypoxia score and Senescore. **(D)** The correlation between angiogenesis score and Senescore. **(E)** Heatmap showing the correlation between predicted immune cell score by CIBERSORT and Senescore. *, p< 0.05; **, p<0.01; ***, p<0.001; ****, p<0.0001.

Based on the results mentioned above, cell senescence-associated subtypes were associated with cell cycle, telomere extension by telomerase, hypoxia, angiogenesis, and immunity. Therefore, the correlations of those scores with the cell senescence-related risk score were analyzed using the rcorr function in the Hmisc package (**Figure 11**).

### Prediction of immunotherapeutic efficacy with the cell senescence-related risk score

**Figure 12:**
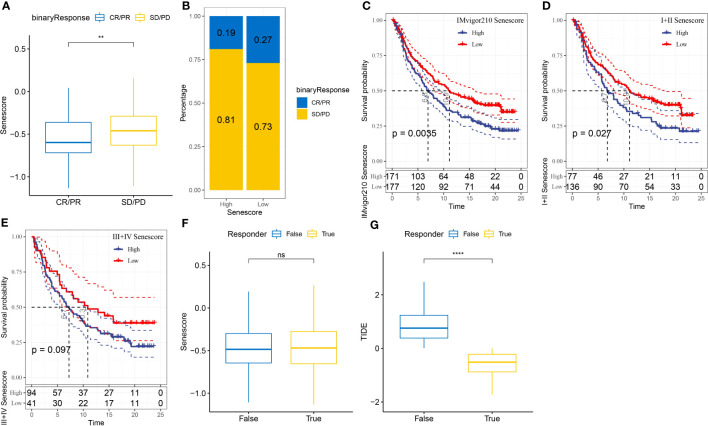
Application of the Senescore in immune therapy. **(A)** Differences in Senescore among immune therapy responders in the IMvigor210 cohort. **(B)** Distribution of immune therapy response among Senescore groups in the IMvigor210 cohort. **(C)** Differences in prognosis among Senescore groups in the IMvigor210 cohort. **(D)** Differences in prognosis among early-stage patients in the Senescore groups of the IMvigor210 cohort. **(E)** Differences in prognosis among late-stage patients in the Senescore groups of the IMvigor210 cohort. **(F)** Differences in Senescore among immune therapy responders analyzed by TIDE in the IMvigor210 cohort. G: Differences in TIDE among immune therapy responders analyzed by TIDE in the IMvigor210 cohort. **, p<0.01. ns, p≥0.05; ****, p<0.0001.

To assess the relevance of the Senescore to immunotherapy, we evaluated the predictive capability of the Senescore for patient response to ICB therapy. In the anti-PD-L1 cohort (IMvigor210 cohort), a high Senescore was associated with a worse prognosis (**Figure 12C**; log-rank test, p< 0.05). Additionally, we found the varied response of 348 patients in the IMvigor210 cohort to PD-L1 blockers, encompassing complete response (CR), partial response (PR), stable disease (SD), and progressive disease (PD). The patients with SD/PD had a higher Senescore versus those with CR/PR (**Figure 12A**). Based on percentage statistics between the low- and high-Senescore groups, significantly better treatment outcomes were noted in patients with a low Senescore (**Figure 12B**). We analyzed survival differences among all samples in the IMvigor210 cohort as well as the survival differences at different Stages. The results showed significant survival differences among Stage I + II samples (**Figure 12D**), but insignificant survival differences between low- and high-Senescore groups in Stage III + IV samples (**Figure 12E**). Particularly, the Senescore exhibited outstanding predictive performance in early-stage clinical samples.

The result from TIDE showed that a higher TIDE prediction score denoted a higher probability of immune escape, indicating a lower probability that the patient might benefit from immunotherapy. Furthermore, the Senescore and TIDE scores were higher in non-responders to immunotherapy, which indirectly suggested that patients with high Senescore were less prone to benefit from immunotherapy (**Figures 12F, G**).

Additionally, we assessed the efficacy of Senescore on other immunotherapy cohorts. Our findings indicate that the GSE135222 cohort shows a different response when compared to the IMvigor210 cohort. Patients with high Senescore performances exhibited enhanced benefits from immunotherapy and sustained better prognoses. Nevertheless, in the GSE78220 immunotherapy cohort, the high and low Senescore divisions did not function as dependable predictive markers for patient outcomes (**Supplementary Figure 3**).

### Correlations of cell senescence-related risk scores and drug sensitivity

**Figure 13:**
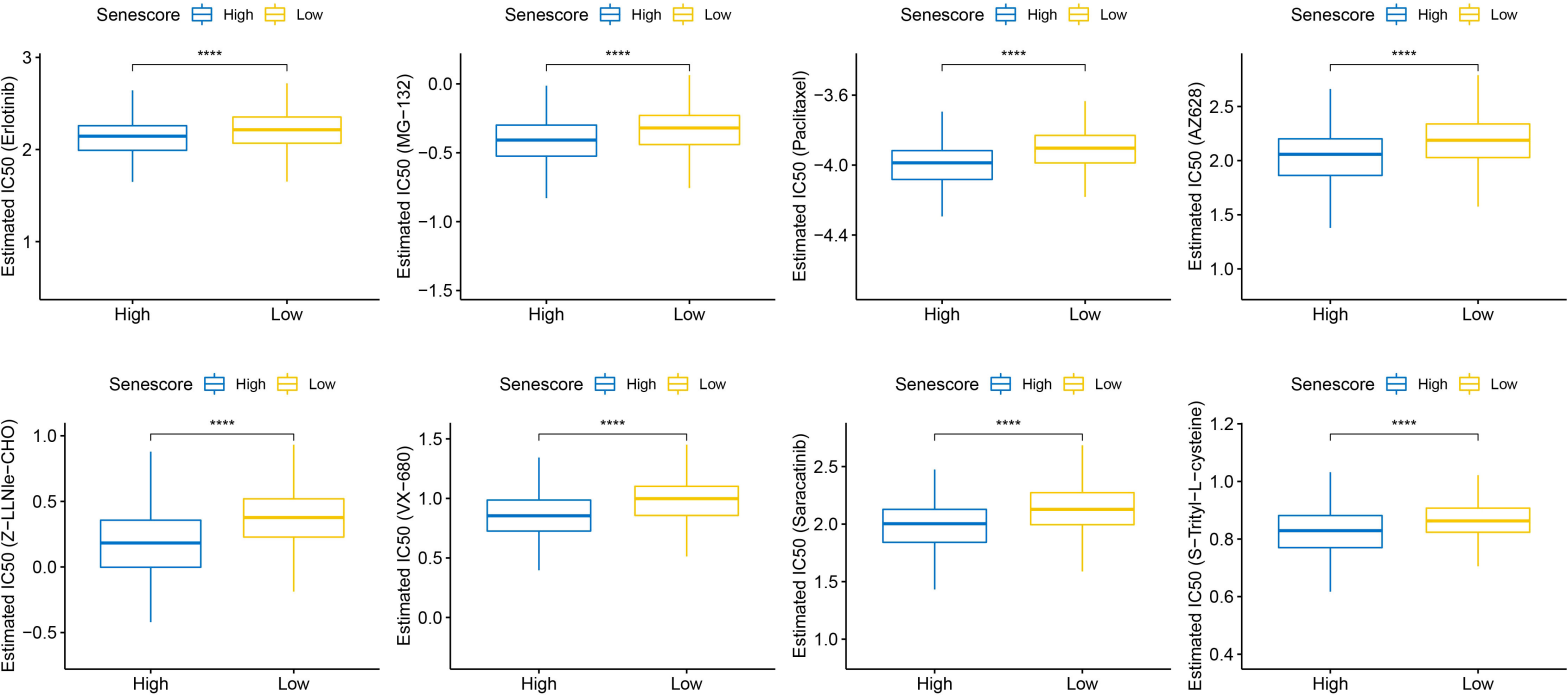
Differential analysis of Senescore and IC50 drug sensitivity. ****, p<0.0001.

This study also compared the response of high- and low-risk populations to conventional chemotherapeutic agents such as Erlotinib, MG-132, and Paclitaxel. The high Senescore group showed a higher sensitivity to the abovementioned agents (**Figure 13**).

### Senescore integrated with clinicopathological features for improved prognostic models and survival prediction

**Figure 14:**
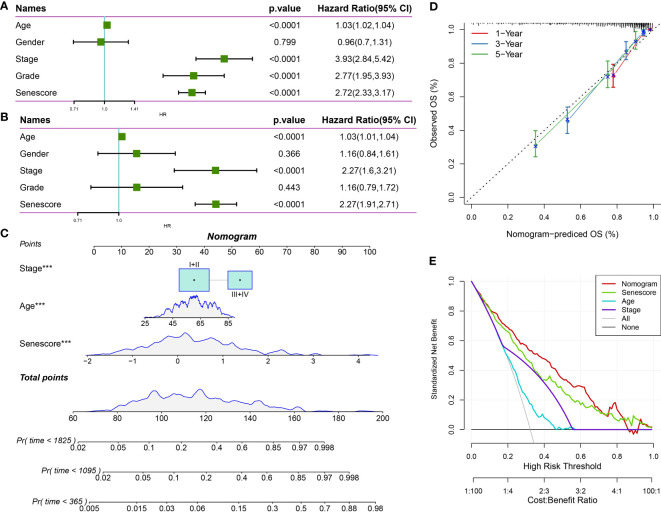
The clinical applications of Senescore. **(A, B)** Univariate and multivariate Cox analyses of Senescore and clinical pathological features; **(C)** Construction of the nomogram model; **(D)** Calibration curves of the nomogram in 1, 3, and 5 years; **(E)** Clinical applications of nomogram model and Senescore. ***, p<0.001.

Through univariate and multivariate Cox regression analyses of the Senescore and clinicopathological characteristics, the Senescore was identified as the most significant prognostic factor (**Figures 14A, B**). To quantify patients’ risk assessment and survival probability, we integrated the Senescore and other clinicopathological characteristics to establish a nomogram (**Figure 14C**). Based on the results. The Senescore exhibited the most significant impact on survival prediction. Furthermore, a calibration curve was utilized for the predictive accuracy assessment. As presented in **Figure 14D**, the calibration curves for the prediction at the three calibration points (1, 3, and 5 years) almost coincided with the standard curves, suggesting the good predictive performance of the nomogram. Also, decision curve analysis (DCA) was carried out to evaluate the reliability of this model. The benefit of either Senescore or nomogram was remarkably higher than that of the extreme curve. Compared to other clinicopathological characteristics, nomogram, and Senescore exhibited more vital survival prediction ability (**Figure 14E**).

## Discussion

Based on single-cell data analysis, this study interpreted the abnormalities in cell senescence-associated pathways within the TME. Meanwhile, we screened cell senescence-associated pathways enriched in tumor tissues in bulk RNA-seq dataset by GSEA and constructed cell senescence-associated subtypes and risk models based on the genes in these pathways. Moreover, this study linked the efficacy of immunotherapy to cell senescence-associated risk scores.

ccRCC is a highly heterogeneous renal tumor developed by different complex epigenetic driving mechanisms and molecular pathways. Due to its malignant progression and high recurrence rate, ccRCC is the deadliest type of renal tumor. Currently, postoperative recovery in patients with ccRCC remains unsatisfactory, and only a few patients may benefit from drug therapy targeting tyrosine kinase inhibitors (TKI) and anti-PD-1 antibodies (16). Biomarkers that can accurately predict prognosis and guide the treatment of ccRCC have not been fully identified and applied clinically. Immunosenescence refers to the age-related decline in immune system function, which can impair the body’s ability to defend against infections, vaccines, and tumors (17). As a result, older individuals are more susceptible to infections and less capable of mounting an effective immune response to diseases. The increased risk of cancer in older adults is of particular concern, as tumor cells often exploit deficiencies in the immune system to evade detection and clearance, making treatment more challenging (18). Consequently, immunosenescence has emerged as a prominent topic in medical research. By exploring the underlying mechanisms of immunosenescence and its impact on human health, researchers hope to develop new strategies to enhance immune function and improve the health and well-being of older individuals. Cellular senescence is a permanent state of the arrest of the cell cycle, and senescent cells/genes have been observed to accumulate during aging and play a role in various tumors (19). Xu et al. constructed a senescence-associated prognostic model significantly correlated with lung adenocarcinoma diagnosis and prognosis (20). In addition, a previous study showed that senescence-associated protein P400 is a prognostic marker for renal cell carcinoma (21). However, senescence characteristics and senescence-associated prognostic models of ccRCC are still rare. The specific molecular markers for ccRCC also need to be further elucidated.

Many studies have attempted to build models based on gene sequencing and clinical data to predict the prognosis of patients with ccRCC (22, 23). However, the clinical application of these models has had little effect. In this study, we used RNA-seq combined with bulk RNA-Seq to analyze the cellular senescence characteristics of ccRCC. Our assessment has recognized IFITM1, SOCS3, DPYSL3, IL20RB, SLC44A4, SEMA3G, ITGA8, PCSK6, ZNF521, and DUSP1 as genes that exhibit noteworthy differential expression in association with senescence in patients with ccRCC. A prognostic model based on these 10 genes is established. Our senescence-related prognostic features showed good performance, with the AUC values of 1-year, 3-year, and 5-year OS predicted by the TCGA database being 0.84, 0.81, and 0.79, respectively. We also used the ICGC dataset to further validate and evaluate the prognosis. The ICGC database predicted the AUC value of 1-year, 3-year, and 5-year OS to be 0.6, 0.69, and 0.7, respectively. The results showed that the senescence-associated prognostic model we constructed could predict the survival rate of ccRCC. Regrettably, extending this gene signature to other types of tumors is unfeasible. Currently, the markers of aging in tumors remain unclear and may vary depending on tumor type and subtype. Each tumor type has distinct biological and genetic characteristics, which could affect the expression of senescence markers in tumor cells. Therefore, a more thorough analysis and evaluation must first be conducted before investigating the applicability of aging markers in specific tumors. In the future, we hope to identify more generalized, broad-spectrum aging markers that can be utilized across different tumor types, facilitating more precise and convenient guidance for studying tumor subtypes.

DUSP1 is a threonine-tyrosine bispecific phosphatase that targets negatively regulating the MAPK signaling pathway (24). Nevertheless, its role in tumorigenesis is controversial. DUSP1 is highly expressed in a range of tumors, including lung, breast, ovarian, gastric, and prostate cancers (25–29), while low expression in HCC (30). Several studies have demonstrated the involvement of DUSP1 in malignant tumor progression through various signaling pathways (31). However, the role of DUSP1 in ccRCC remains unknown. The results of this study indicate that DUSP1 is one of the essential regulatory genes of senescence characteristics in ccRCC. The results of specific staining experiments demonstrated that DUSP1 plays an essential role in the senescence of renal clear cell carcinoma cells by effectively inhibiting the generation of senescent cells. *In vitro* experiments, we found that DUSP1 knockdown significantly promoted the proliferation, migration, and invasion of renal carcinoma cells. Targeting DUSP1 may serve as a potential “senescence biomarker” for predicting clinical outcomes in patients with ccRCC.

The tumor microenvironment (TME) comprises various types of cells, including tumor cells, inflammatory cells, immune cells, stromal stem cells, endothelial cells, and tumor-associated fibroblasts, which play essential roles in the proliferation and drug resistance of tumor cells. Cellular senescence in TME can trigger immune cell infiltration and promote tumor progression (32, 33). This study found that T cells, B cells, mast cells, macrophage, fibroblast, and endothelial cells were significant tumor-infiltrating cell clusters compared to adjacent normal tissues. We also found that fibroblast senescence-related pathway scores were higher in ccRCC than adjacent normal tissue cells. Tumor fibroblasts are the primary source of tumor extracellular matrix (ECM) (34). Shi et al. demonstrated that the recruitment of cancer-associated fibroblasts (CAFs) in the ccRCC microenvironment occurs through interaction with malignant proximal renal tubular epithelial cells (PTEC) (35). Peng et al. showed that infiltrating CAFs could reduce CD8^+^ T cell infiltration in the TME of ccRCC by secreting galactolectin 73 (Gal1) (36). The study found that CAFs can provide metabolic support to cancer cells by releasing alanine and deoxycytidine, thereby enhancing chemotherapy drug resistance (37, 38). Extracellular vesicles from CAFs have also been shown to contain various surface proteins (CD105, Hsp70, TGF-β1, etc.) and metabolites (lactate, amino acids, lipids, etc.), which can affect tumor progression and drug resistance (39). CAFs may represent a novel therapeutic target to combat resistance to ccRCC treatment. Targeting CAFs with immunotherapy is also emerging as an effective treatment for ccRCC.

This study investigated the correlation between multiple molecular characteristics, such as telomerase, EMT, and angiogenesis, representing different physiological processes and cell senescence scores. Furthermore, the study explored the relationship between immune infiltration levels in the tumor microenvironment and cell aging scores. Although specific impacts of each characteristic on immune infiltration have been reported, for instance, the telomerase catalytic subunit (TERT) activates endogenous retroviruses to promote the formation of a tumor immune suppression microenvironment (40). At the same time, epithelial-mesenchymal transition (EMT) plays a vital role in immune evasion and tumor immune suppression. The EMT score may be a predictive biomarker for clinical response to immune checkpoint blockade. In addition, tumor angiogenesis is associated with the infiltration of different immune cell types in the TME, which could affect the response of tumors to immunotherapy. These results suggest that cell aging affects immune infiltration through multiple molecular mechanisms. However, further research is necessary to understand the precise impact of cell aging on immune infiltration. Through correlational analysis of these results, it is expected to link cell senescence, immune infiltration, and molecular features, providing a comprehensive understanding and assistance for ccRCC treatment.

Furthermore, this study explored the potential use of risk scores associated with cellular senescence in immune therapy by linking them to its effectiveness. We conducted an effective analysis of the correlation and difference between the senescence score and the infiltration level of immune cells, which is critical when predicting the results of immune therapy using risk scores associated with cellular senescence. Notably, both significant differences in infiltration levels of macrophages among different subtypes and a significant correlation between senescence score and macrophages were shown by the results. M0 macrophages are undifferentiated precursor cells that differentiate into either M1 (pro-inflammatory) or M2 (anti-inflammatory) macrophages according to the needs of the TME. Although we cannot accurately evaluate the precise effects of cell senescence signatures on immune cells, we hypothesize that genes in the model may facilitate the polarization of M0 macrophages toward tumor-promoting M2 macrophages during polarization. For example, SOCS3 plays an important role in the polarization of M2 macrophages (10), but the roles of other genes in macrophage polarization remain unclear. Our main goal is to guide immune therapy for patients by reflecting the infiltration of immune cells in complex TMEs and highly heterogeneous backgrounds using senescence scores. Overall, these results suggest that it is possible to evaluate the infiltration of immune cells in the TME or the molecular characteristics of different tumor patients by using senescence scores. Senescence scores can be used as a useful marker to evaluate the infiltration level of immune cells effectively and conveniently in TME.

Although current research provides a relatively clear understanding of the senescence features in ccRCC and enhances our understanding of the role of cellular senescence in ccRCC, some limitations still cannot be ignored. Although this study validated the results using multiple ccRCC patient cohorts, the limited sample size may not fully represent the characteristics of the entire ccRCC patient population. Additionally, the data used in this study may come from limited datasets or databases and, therefore, may not fully cover all relevant information. Finally, this study did not consider potential external factors such as environmental factors and lifestyle, which may impact the conclusions drawn in this study. In summary, this study uncovered abnormal senescence-related pathways in the TME of ccRCC using a combination of single-cell data and bulk RNA-seq. Based on these dysregulated aging signaling pathways, senescence-related subtypes and risk models were created, which offer new methods to evaluate prognosis, guide clinical drug selection, and assess immune therapy response in ccRCC patients. Targeting essential senescence-related genes may lead to a new understanding of the molecular mechanisms of aging in ccRCC and their clinical applications.

## Data availability statement

The datasets presented in this study can be found in online repositories. The names of the repository/repositories and accession number(s) can be found within the article/**Supplementary Materials**.

## Ethics statement

All the procedures were performed in compliance with the National Institutes of Health Guidelines for the Care and Use of Laboratory Animals and ARRIVE Guidelines.

## Author contributions

QG and FL designed the experiments. QG, FL, YJ and JX performed the experiments, QG and JG wrote the manuscript. All authors contributed to the article and approved the submitted version.

## References

[1] Padala SA Barsouk A Thandra KC Saginala K Mohammed A Vakiti A. Epidemiology of renal cell carcinoma. World J Oncol (2020) 11(3):79–87. doi: 10.14740/wjon1279 32494314 PMC7239575

[2] Jonasch E Walker CL Rathmell WK. Clear cell renal cell carcinoma ontogeny and mechanisms of lethality. Nat Rev Nephrol (2021) 17(4):245–61. doi: 10.1038/s41581-020-00359-2 PMC8172121 33144689

[3] Tran J Ornstein MC. Clinical review on the management of metastatic renal cell carcinoma. JCO Oncol Pract (2022) 18(3):187–96. doi: 10.1200/op.21.00419 34529499

[4] Raghubar AM Roberts MJ Wood S Healy HG Kassianos AJ Mallett AJ. Cellular milieu in clear cell renal cell carcinoma. Front Oncol (2022) 12:943583. doi: 10.3389/fonc.2022.943583 36313721 PMC9614096

[5] Klatte T Rossi SH Stewart GD. Prognostic factors and prognostic models for renal cell carcinoma: a literature review. World J Urol (2018) 36(12):1943–52. doi: 10.1007/s00345-018-2309-4 29713755

[6] Calcinotto A Kohli J Zagato E Pellegrini L Demaria M Alimonti A. Cellular senescence: aging, cancer, and injury. Physiol Rev (2019) 99(2):1047–78. doi: 10.1152/physrev.00020.2018 30648461

[7] Sieben CJ Sturmlechner I van de Sluis B van Deursen JM. Two-step senescence-focused cancer therapies. Trends Cell Biol (2018) 28(9):723–37. doi: 10.1016/j.tcb.2018.04.006 PMC6102047 29776716

[8] Kuilman T Michaloglou C Vredeveld LC Douma S van Doorn R Desmet CJ. Oncogene-induced senescence relayed by an interleukin-dependent inflammatory network. Cell (2008) 133(6):1019–31. doi: 10.1016/j.cell.2008.03.039 18555778

[9] Ziegenhain C Vieth B Parekh S Reinius B Guillaumet-Adkins A Smets M. Comparative analysis of single-cell rna sequencing methods. Mol Cell (2017) 65(4):631–43.e4. doi: 10.1016/j.molcel.2017.01.023 28212749

[10] Ren J Zhu B Gu G Zhang W Li J Wang H. Schwann cell-derived exosomes containing mfg-E8 modify Macrophage/Microglial polarization for attenuating inflammation via the Socs3/Stat3 pathway after spinal cord injury. Cell Death Dis (2023) 14(1):70. doi: 10.1038/s41419-023-05607-4 36717543 PMC9887051

[11] Sanchez-Vega F Mina M Armenia J Chatila WK Luna A La KC. Oncogenic signaling pathways in the cancer genome atlas. Cell (2018) 173(2):321–37.e10. doi: 10.1016/j.cell.2018.03.035 29625050 PMC6070353

[12] Liu F Wang P Sun W Jiang Y Gong Q. Identification of ligand-receptor pairs associated with tumour characteristics in clear cell renal cell carcinoma. Front Immunol (2022) 13:874056. doi: 10.3389/fimmu.2022.874056 35734169 PMC9207243

[13] Gong Q Guo Z Sun W Du X Jiang Y Liu F. Cx3cl1 promotes cell sensitivity to ferroptosis and is associated with the tumor microenvironment in clear cell renal cell carcinoma. BMC Cancer (2022) 22(1):1184. doi: 10.1186/s12885-022-10302-2 36397015 PMC9670481

[14] Gong Q Jiang Y Pan X You Y. Fractalkine aggravates lps-induced macrophage activation and acute kidney injury via Wnt/B-catenin signalling pathway. J Cell Mol Med (2021) 25(14):6963–75. doi: 10.1111/jcmm.16707 PMC8278080 34101346

[15] Wang L Lankhorst L Bernards R. Exploiting senescence for the treatment of cancer. Nat Rev Cancer (2022) 22(6):340–55. doi: 10.1038/s41568-022-00450-9 35241831

[16] Yao X Tan J Lim KJ Koh J Ooi WF Li Z. Vhl deficiency drives enhancer activation of oncogenes in clear cell renal cell carcinoma. Cancer Discovery (2017) 7(11):1284–305. doi: 10.1158/2159-8290.Cd-17-0375 28893800

[17] Daste A Domblides C Gross-Goupil M Chakiba C Quivy A Cochin V. Immune checkpoint inhibitors and elderly people: a review. Eur J Cancer (2017) 82:155–66. doi: 10.1016/j.ejca.2017.05.044 28689093

[18] White MC Holman DM Goodman RA Richardson LC. Cancer risk among older adults: time for cancer prevention to go silver. Gerontol (2019) 59(Supplement_1):S1–6. doi: 10.1093/geront/gnz038 PMC6738561 31511747

[19] Pérez-Mancera PA Young AR Narita M. Inside and out: the activities of senescence in cancer. Nat Rev Cancer (2014) 14(8):547–58. doi: 10.1038/nrc3773 25030953

[20] Xu Q Chen Y. An aging-related gene signature-based model for risk stratification and prognosis prediction in lung adenocarcinoma. Front Cell Dev Biol (2021) 9:685379. doi: 10.3389/fcell.2021.685379 34277626 PMC8283194

[21] Macher-Goeppinger S Bermejo JL Schirmacher P Pahernik S Hohenfellner M Roth W. Senescence-associated protein P400 is a prognostic marker in renal cell carcinoma. Oncol Rep (2013) 30(5):2245–53. doi: 10.3892/or.2013.2698 23982490

[22] Su C Lv Y Lu W Yu Z Ye Y Guo B. Single-cell rna sequencing in multiple pathologic types of renal cell carcinoma revealed novel potential tumor-specific markers. Front Oncol (2021) 11:719564. doi: 10.3389/fonc.2021.719564 34722263 PMC8551404

[23] Zhong W Zhang F Huang C Lin Y Huang J. Identification of epithelial-mesenchymal transition-related lncrna with prognosis and molecular subtypes in clear cell renal cell carcinoma. Front Oncol (2020) 10:591254. doi: 10.3389/fonc.2020.591254 33324563 PMC7724112

[24] Low HB Zhang Y. Regulatory roles of mapk phosphatases in cancer. Immune Netw (2016) 16(2):85–98. doi: 10.4110/in.2016.16.2.85 27162525 PMC4853501

[25] Bang YJ Kwon JH Kang SH Kim JW Yang YC. Increased mapk activity and mkp-1 overexpression in human gastric adenocarcinoma. Biochem Biophys Res Commun (1998) 250(1):43–7. doi: 10.1006/bbrc.1998.9256 9735328

[26] Chattopadhyay S Machado-Pinilla R Manguan-García C Belda-Iniesta C Moratilla C Cejas P. Mkp1/Cl100 controls tumor growth and sensitivity to cisplatin in non-Small-Cell lung cancer. Oncogene (2006) 25(23):3335–45. doi: 10.1038/sj.onc.1209364 16462770

[27] Gil-Araujo B Toledo Lobo MV Gutiérrez-Salmerón M Gutiérrez-Pitalúa J Ropero S Angulo JC. Dual specificity phosphatase 1 expression inversely correlates with nf-Kb activity and expression in prostate cancer and promotes apoptosis through a P38 mapk dependent mechanism. Mol Oncol (2014) 8(1):27–38. doi: 10.1016/j.molonc.2013.08.012 24080497 PMC5528511

[28] Shi YY Small GW Orlowski RZ. Proteasome inhibitors induce a P38 mitogen-activated protein kinase (Mapk)-dependent anti-apoptotic program involving mapk phosphatase-1 and akt in models of breast cancer. Breast Cancer Res Treat (2006) 100(1):33–47. doi: 10.1007/s10549-006-9232-x 16807678

[29] Wang HY Cheng Z Malbon CC. Overexpression of mitogen-activated protein kinase phosphatases Mkp1, Mkp2 in human breast cancer. Cancer Lett (2003) 191(2):229–37. doi: 10.1016/s0304-3835(02)00612-2 12618338

[30] Du Y Lu S Ge J Long D Wen C Tan S. Rock2 disturbs Mkp1 expression to promote invasion and metastasis in hepatocellular carcinoma. Am J Cancer Res (2020) 10(3):884–96. PMC7136912 32266097

[31] Shen J Zhou S Shi L Liu X Lin H Yu H. Dusp1 inhibits cell proliferation, metastasis and invasion and angiogenesis in gallbladder cancer. Oncotarget (2017) 8(7):12133–44. doi: 10.18632/oncotarget.14815 PMC5355331 28129656

[32] Song S Tchkonia T Jiang J Kirkland JL Sun Y. Targeting senescent cells for a healthier aging: challenges and opportunities. Adv Sci (Weinh) (2020) 7(23):2002611. doi: 10.1002/advs.202002611 33304768 PMC7709980

[33] Sarode P Schaefer MB Grimminger F Seeger W Savai R. Macrophage and tumor cell cross-talk is fundamental for lung tumor progression: we need to talk. Front Oncol (2020) 10:324. doi: 10.3389/fonc.2020.00324 32219066 PMC7078651

[34] Bond KH Chiba T Wynne KPH Vary CPH Sims-Lucas S Coburn JM. The extracellular matrix environment of clear cell renal cell carcinoma determines cancer associated fibroblast growth. Cancers (Basel) (2021) 13(23):5873. doi: 10.3390/cancers13235873 PMC8657052 34884982

[35] Shi Y Zhang Q Bi H Lu M Tan Y Zou D. Decoding the multicellular ecosystem of vena caval tumor thrombus in clear cell renal cell carcinoma by single-cell rna sequencing. Genome Biol (2022) 23(1):87. doi: 10.1186/s13059-022-02651-9 35361264 PMC8969307

[36] Peng YL Xiong LB Zhou ZH Ning K Li Z Wu ZS. Single-cell transcriptomics reveals a low Cd8(+) T cell infiltrating state mediated by fibroblasts in recurrent renal cell carcinoma. J Immunother Cancer (2022) 10(2):e004206. doi: 10.1136/jitc-2021-004206 PMC8819783 35121646

[37] Dalin S Sullivan MR Lau AN Grauman-Boss B Mueller HS Kreidl E. Deoxycytidine release from pancreatic stellate cells promotes gemcitabine resistance. Cancer Res (2019) 79(22):5723–33. doi: 10.1158/0008-5472.Can-19-0960 PMC7357734 31484670

[38] Parker SJ Amendola CR Hollinshead KER Yu Q Yamamoto K Encarnación-Rosado J. Selective alanine transporter utilization creates a targetable metabolic niche in pancreatic cancer. Cancer Discovery (2020) 10(7):1018–37. doi: 10.1158/2159-8290.Cd-19-0959 PMC7334074 32341021

[39] Kahlert C Kalluri R. Exosomes in tumor microenvironment influence cancer progression and metastasis. J Mol Med (Berl) (2013) 91(4):431–7. doi: 10.1007/s00109-013-1020-6 PMC4073669 23519402

[40] Mao J Zhang Q Wang Y Zhuang Y Xu L Ma X. Tert activates endogenous retroviruses to promote an immunosuppressive tumour microenvironment. EMBO Rep (2022) 23(4):e52984. doi: 10.15252/embr.202152984 35107856 PMC8982579

