# Supplementary figures and images for: Integrating scRNA and bulk-RNA sequencing develops a cell senescence signature for analyzing tumor heterogeneity in clear cell renal cell carcinoma
Source: Front Immunol. 2023 Jul 12;14:1199002. doi: 10.3389/fimmu.2023.1199002 (PMC10370498; doi:10.3389/fimmu.2023.1199002)

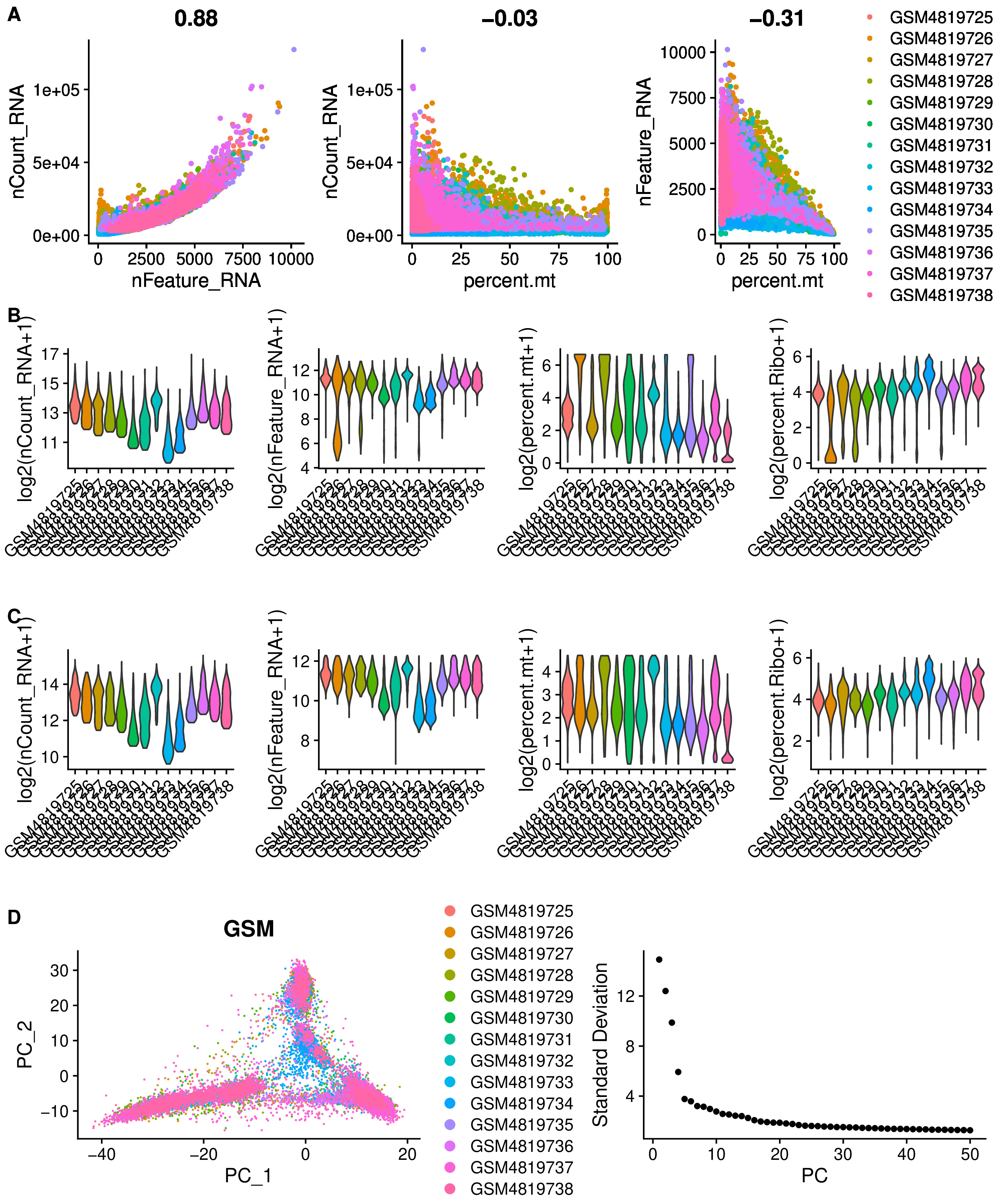

Supplement: Supplementary file 1 [file Image_1.jpeg]

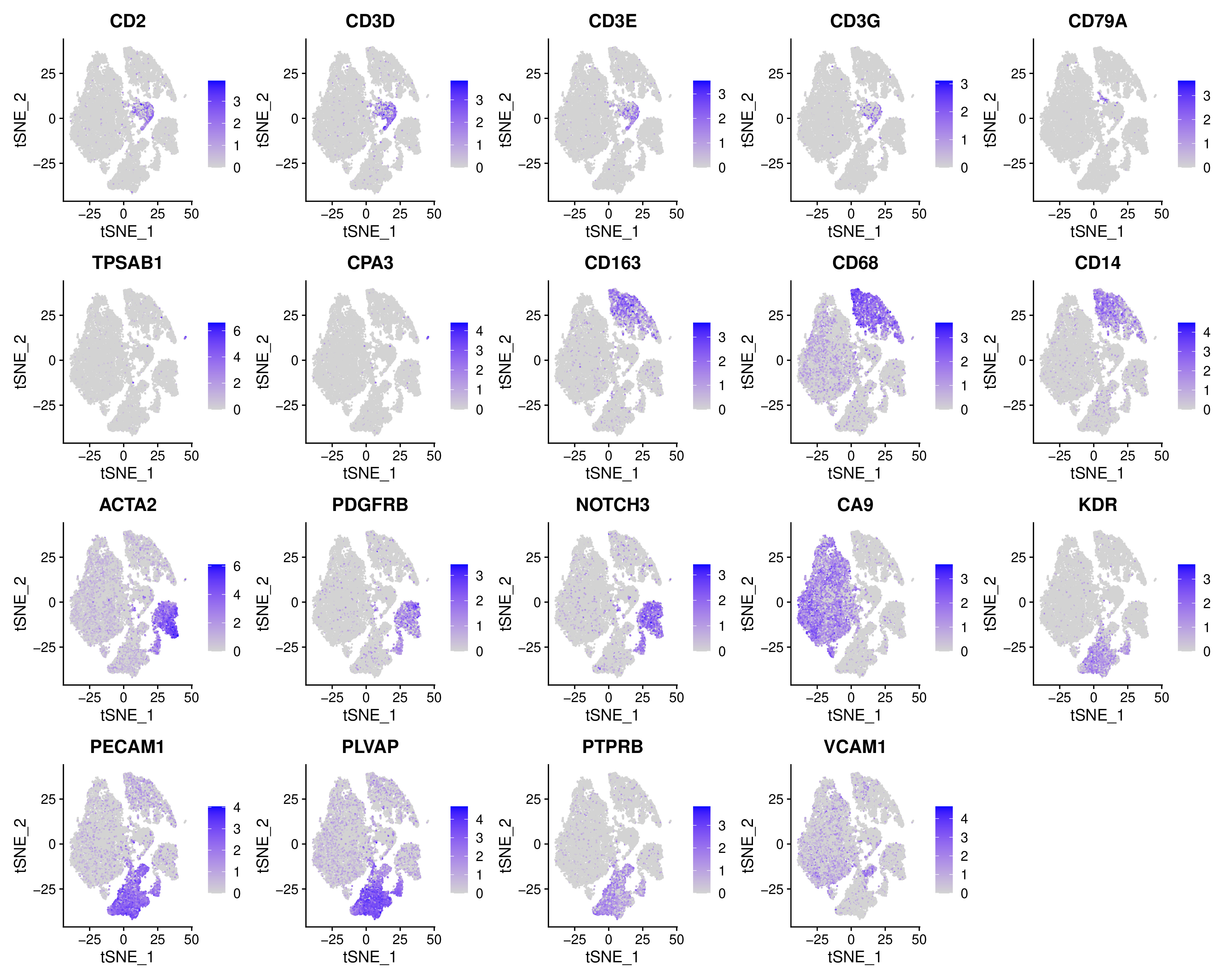

Supplement: Supplementary file 2 [file Image_2.jpeg]

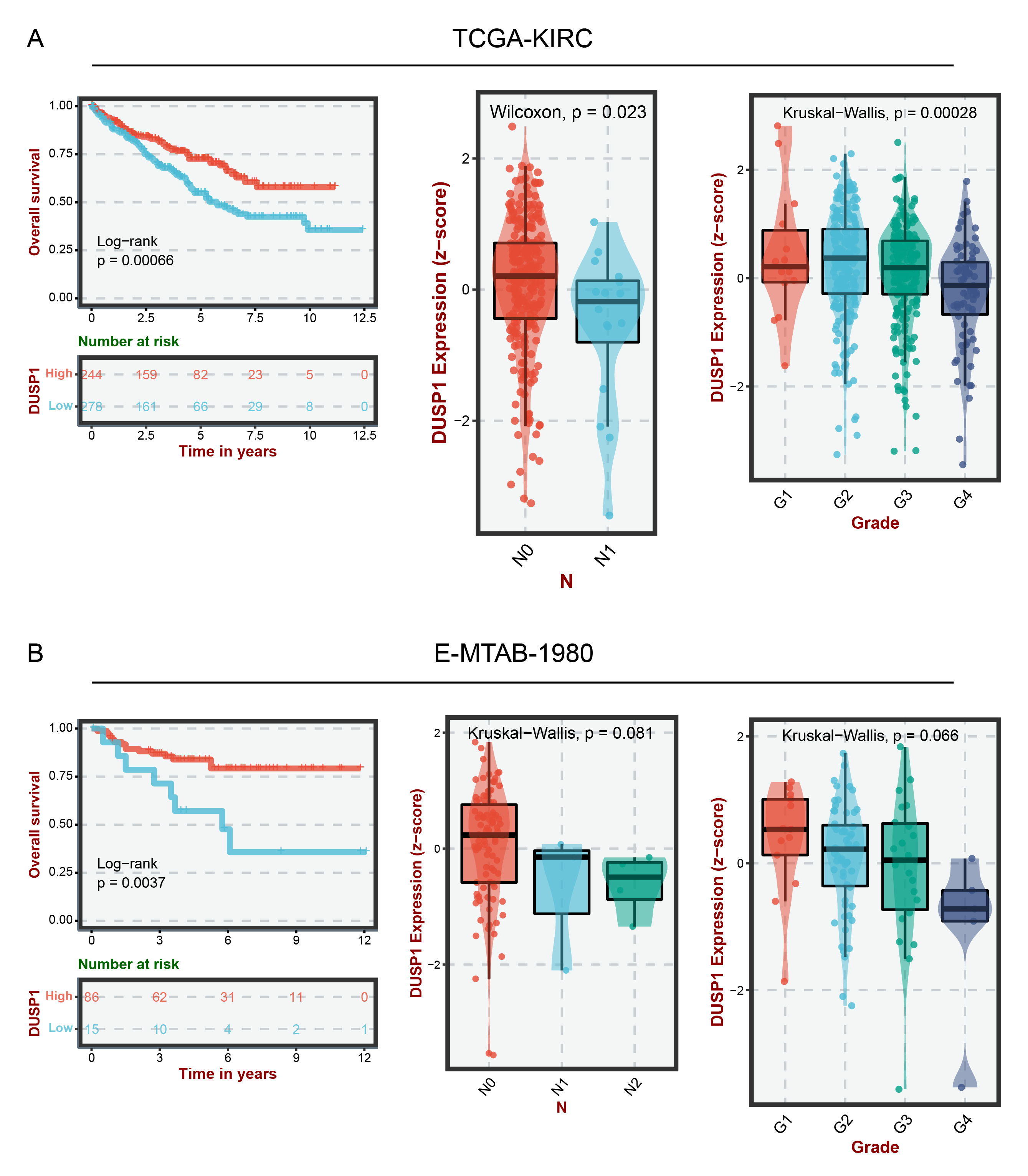

Supplement: Supplementary file 3 [file Image_3.jpeg]

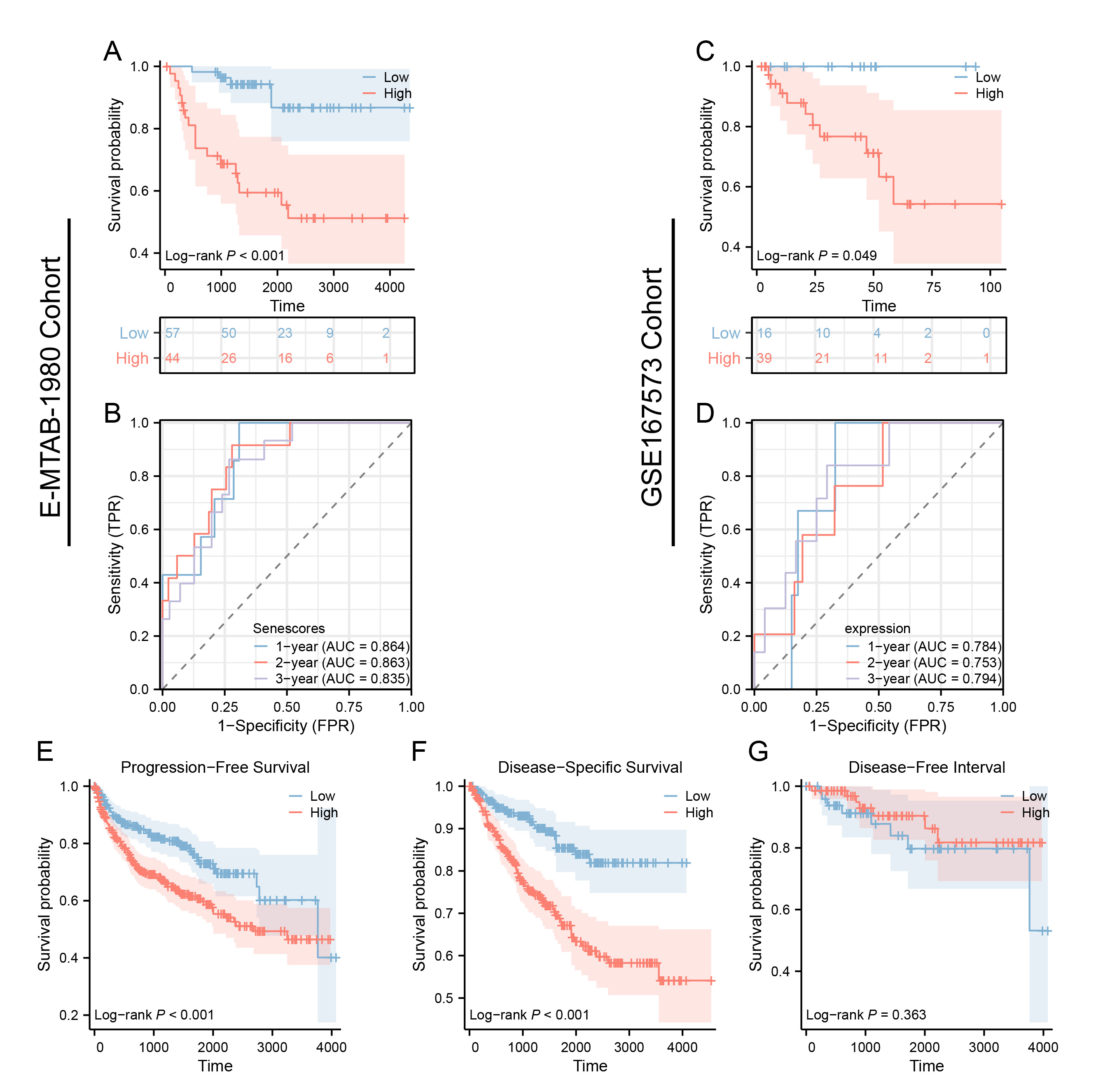

Supplement: Supplementary file 4 [file Image_4.jpeg]

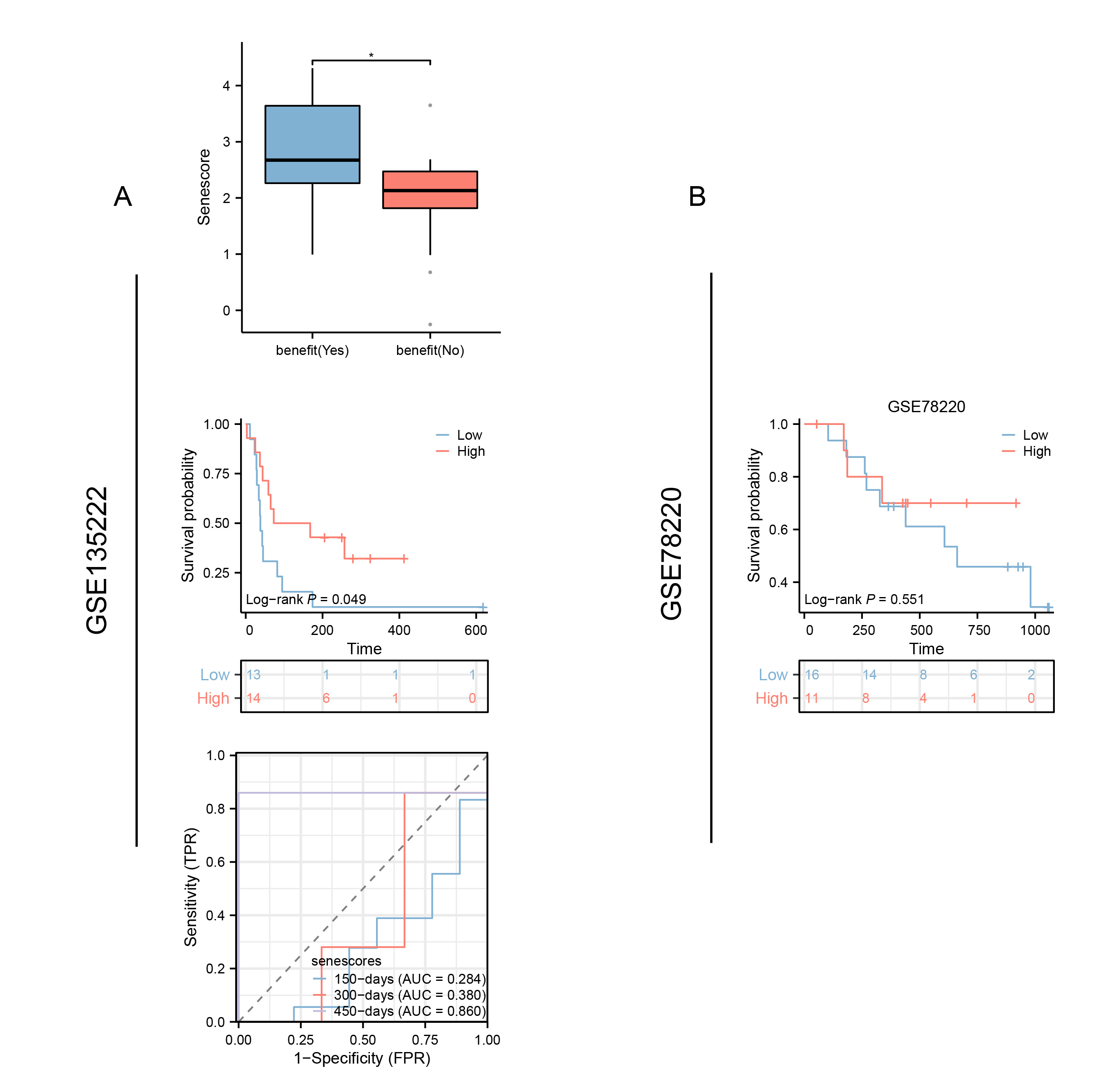

Supplement: Supplementary file 5 [file Image_5.jpeg]
