# Supplementary material for: Integrating scRNA and bulk-RNA sequencing develops a cell senescence signature for analyzing tumor heterogeneity in clear cell renal cell carcinoma
Source: Front Immunol. 2023 Jul 12;14:1199002. doi: 10.3389/fimmu.2023.1199002 (PMC10370498; doi:10.3389/fimmu.2023.1199002)
Supplement: Supplementary file 7 [file Table_2.docx]

https://www.jianguoyun.com/p/DUAnW4QQqdzxCRjN3oYFIAA
